# Similarities between Tumour Immune Response and Chronic Wound Microenvironment: Influence of Mesenchymal Stromal/Stem Cells

**DOI:** 10.1155/2021/6649314

**Published:** 2021-03-29

**Authors:** Kimberly Thando Peta, Melvin Anyasi Ambele, Michael Sean Pepper

**Affiliations:** ^1^Institute for Cellular and Molecular Medicine, Department of Immunology, SAMRC Extramural Unit for Stem Cell Research and Therapy, Faculty of Health Sciences, University of Pretoria, Private Bag X323, Arcadia, 0007, South Africa; ^2^Department of Oral Pathology and Oral Biology, School of Dentistry, Faculty of Health Sciences, University of Pretoria, PO Box 1266, Pretoria 0001, South Africa

## Abstract

Tumours are characterized by a state of chronic inflammation and are regarded as wounds that never heal. Mesenchymal stromal/stem cells (MSCs) are being considered as a possible treatment option. While MSCs can regulate the immune system, migrate to sites of inflammation, and are naturally immune-privileged, there have been contradictory reports on the role of these cells in the tumour microenvironment (TME). Some studies have suggested that MSCs promote tumourigenesis while others have suggested the contrary. To better evaluate the role of MSCs in the TME, it may be helpful to understand the role of MSCs in chronic wounds. Here, we discuss the role of MSCs in chronic wounds and extrapolate this to the TME. Chronic wounds are stuck in the inflammatory phase of wound healing, while in the case of the TME, both the inflammatory and proliferative phases are exploited. MSCs in chronic wounds promote a switch in macrophage phenotype from proinflammatory (M1) to anti-inflammatory (M2), thereby suppressing T, B, and natural killer cells, consequently promoting wound healing. In the case of the TME, MSCs are reported to promote tumorigenesis by suppressing T, B, and natural killer cells in addition to dendritic cells, cytotoxic T cells, and Th1-associated cytokines, thereby promoting tumour growth. Some studies have however suggested that MSCs inhibit tumourigenesis, depending on the source of the MSCs and the specific mediators involved. Therefore, the role of MSCs in the TME appears to be complex and may result in variable outcomes. Compelling evidence to suggest that MSCs are an effective treatment option against tumour progression is lacking.

## 1. Introduction

Mesenchymal stromal/stem cells (MSCs) are multipotent cells that can be differentiated into bone, muscle, cartilage, adipose, and other tissues of mesodermal origin [1, 2]. MSCs are crucial for maintaining tissue homeostasis including for example musculoskeletal tissue and fracture repair [3]. Some characteristics of cultured MSCs include plastic adherence, differentiation *in vitro*, and the differential expression of specific cell surface markers. MSCs are positive for CD105, CD73, and CD90 and negative for CD45, CD34, and CD14 or 11b, CD79, or CD19, and HLA-DR [4]. MSCs from different sources such as bone marrow (BM-MSCs) and adipose tissue MSCs (AT-MSCs) secrete cytokines and chemokines. They also express genes such as *Sox2*, *Oct4*, *p53*, and *c-myc* albeit at different levels [5]. BM-MSCs have different subpopulations with some expressing surface antigens that are not expressed by others. This differential expression may be due to the influence of cell culture conditions [6]. Perinatal MSCs are derived from the umbilical cord, amniotic membrane, maternal decidua, and chorionic villi [7]. Although MSCs derived from umbilical cord blood (UCB) and umbilical cord (UC) are similar, their gene expression profiles vary [8]. Some genes are highly or exclusively expressed by UC such as *NRP*, *SYNP02*, *NPY*, and *CDH2*, which may be related to synaptic transmission or neurogenesis, while UCB exclusively expresses osteoblast-specific factor and osteopontin [8]. MSCs from various sources therefore share common phenotypic properties even though they have different genetic and cytokine profiles [9]. These differences should be considered when using MSCs for research or clinical applications [8]. In this review, the source of MSCs is not mentioned but rather MSCs are spoken of generally based on their shared characteristics.

MSCs migrate to sites of injury where they interact both with the innate and adaptive immune systems leading to tissue repair and regeneration [10, 11]. There is increasing evidence from preclinical research and clinical trials that MSC-based cellular therapies may soon become part of routine medical care. This is because MSCs can differentiate into multiple lineages, secrete cytokines and growth factors, migrate to sites of injury and inflammation, promote cell proliferation and migration, and are considered immune-privileged and lack costimulatory molecules [1, 9, 12]. However, recent evidence has suggested that MSCs do invoke an immune response [13]. The ability of MSCs to regulate inflammatory and immune responses is central to their role in wound healing, particularly in chronic wounds [14, 15].

Cancer has been described as a “wound that never heals” [16]. Several studies have reported that MSCs migrate to tumour sites and promote tumorigenesis and metastasis [7, 17–21], while others have suggested the contrary [22–24]. The exact role of MSCs in the tumour microenvironment (TME) therefore remains poorly understood. We propose that understanding the role of MSCs in chronic wounds, particularly how MSCs influence the immune response, may shed light on the function of MSCs in the TME. This narrative review will therefore address how MSCs influence the immune response in chronic wounds and the TME.

### 1.1. Immune Response in Acute Wounds

Acute wound healing involves an acute inflammatory response comprised of four phases: haemostasis, inflammation, proliferation, and tissue remodelling [25, 26]. Proper wound healing requires the correct functioning of proinflammatory (M1) and anti-inflammatory (M2) macrophages, as well as cytokines, growth factors, proteases, and extracellular and cellular elements [27]. Of particular importance, M1 macrophages are responsible for phagocytosing dead cells, where apoptosis is induced by natural killer (NK) cells, while M2 macrophages support wound closure and angiogenesis [28].

Macrophages and keratinocytes secrete chemoattractant MCP-1, which recruits mast cells (MCs) to acute wound sites to release proinflammatory mediators including histamine, IL-6, IL-8, and VEGF [29]. These proinflammatory mediators facilitate the migration of neutrophils and monocytes to the wound site and increase vasodilation and endothelial permeability [29]. B and T cells are also crucial for wound healing. Nishio et al. have shown that splenectomised nude mice with acute wounds have fewer B and T cells in the wound area but more antibodies bound to damaged tissue; reintroducing B cells isolated from the spleen led to wound healing [30].

**Figure 1:**
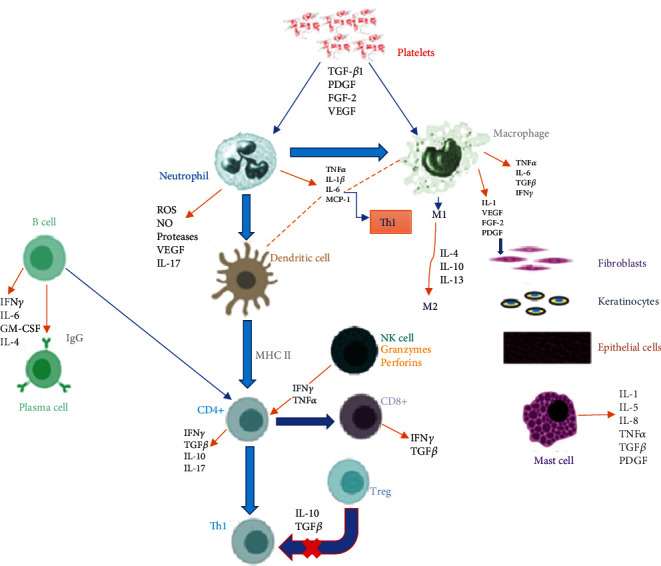
Summary of pro- and anti-inflammatory cells and mediators involved in acute wound healing. During haemostasis, platelets are the first to arrive in the wound microenvironment and secrete TGF-*β*1, PDGF, FGF-2, and VEGF. These cytokines activate and recruit neutrophils and macrophages [25, 43]. During the inflammatory phase, neutrophils secrete ROS, NO, proteases, VEGF, and IL-17 that eliminate pathogens and microbes and promote neutrophil apoptosis [43, 44]. Neutrophils also secrete TNF*α*, IL-1*β*, and IL-6 and MCP-1 that attract monocytes, dendritic cells, and activate T cells causing a Th1 proinflammatory response [33, 45]. After neutrophil apoptosis, macrophages undergo efferocytosis [46]. M1 macrophages secrete IL-1, VEGF, FGF-2, and PDGF that promote the proliferation of fibroblasts, keratinocytes, and epithelial cells. In early wound healing, macrophages secrete TNF*α*, IL-6, and TGF*β* [45]. Macrophages and NK cells secrete IFN-*γ* that attract T cells to the wound site and increase their proliferation [33, 43]. NK cells appear early in wounds and secrete IFN-*γ* and TNF*α* that increase the proliferation of CD4+ T cells. NK cells also release perforins and granzymes that are cytotoxic to infected cells [47]. Mast cells secrete inflammatory cytokines such as IL-1, IL-5, IL-8, TNF*α*, TGF*β*, and PDGF and granules [14, 48]. During the proliferative and remodelling phases, IL-4, IL-10, and IL-13 induce the transition of M1 to M2 macrophages [43, 47]. Dendritic cells act as APCs to CD4+ T cells via MHC II and costimulatory molecules [49]. Once activated, CD4+ and CD8+ T cells migrate to the wound site and secrete IFN-*γ*, TGF*β*, IL-10, IL-17, IL-22, and TGF*β* [33, 50, 51]. B cells accumulate at the wound site and differentiate into plasma cells that release IgG antibodies that aggravate inflammation. B cells act as APCs encouraging T cell activation and secrete IL-6, IFN*γ*, GM-CSF, and IL-4 [52]. Tregs maintain homeostasis and secrete anti-inflammatory cytokines like IL-10 and TGF*β* that inhibit Th1 cells activity (indicated with an X) [53]. Key: TGF-*β*: transforming growth factor-beta; PDGF: platelet-derived growth factor; FGF: fibroblast growth factor; VEGF: vascular endothelial growth factor; ROS: reactive oxygen species; NO: nitric oxide; IL: interleukin; TNF*α*: tumour necrosis factor-alpha; MCP-1: monocyte chemoattractant protein-1; Th: T helper; IFN-*γ*: interferon-gamma; M1: proinflammatory macrophage; M2: anti-inflammatory macrophage; APC: antigen-presenting cell; NK: natural killer; MHC: major histocompatibility complex; IgG: immunoglobulin G; GM-CSF: granulocyte-macrophage colony-stimulating factor.

In acute wound healing, the transition from a pro- to an anti-inflammatory response depends on the presence of foxp3-expressing regulatory T cells (Treg) [31]. Tregs contribute to wound healing in two ways: immune-mediated and non-immune-mediated. The immune-mediated mechanism involves Tregs suppressing proinflammatory stimuli by activating TGF-*β* and secreting IL-10. The non-immune-mediated mechanism involves Tregs that secrete the epidermal growth factor-like growth factor amphiregulin. Amphiregulin stimulates cell differentiation in injured tissue leading to tissue homeostasis, restoration and wound healing [31]. A more elaborate representation of pro- and anti-inflammatory cells and mediators involved in acute wound healing is shown on Figure 1.

### 1.2. Immune Response in Chronic Wounds

Chronic wounds fail to heal when one or more of the wound-healing phases is disrupted [27] resulting in overlapping inflammatory and proliferative phases [32]. The proinflammatory state is characterised by impaired function of neutrophils, macrophages, B cells, T cells [27], and the persistence of these cells in the wound milieu.

Certain factors in chronic wounds such as iron overload may prolong the presence of M1 macrophages, which are responsible for inciting an inflammatory response, stalling resolution of the inflammatory phase, and impairing the function of M2 macrophages [28]. Stalling the resolution of the inflammatory phase is linked to the inability of M1 macrophages to phagocytize dead neutrophils which accumulate at the wound site, thereby promoting the persistence of an inflammatory environment [32]. The build-up of neutrophils at chronic wound sites is therefore associated with a delayed transition from M1 to M2 macrophages, leading to unresolved inflammation. Delayed wound healing causes an exaggerated immune response that leads to further tissue damage instead of restoration. Inflammation in distant locations may also be induced by reverse transendothelial migration (rTEM) which occurs as a result of neutrophils that migrate away from the wound site back into circulation [33].

NK cells are rare or depleted at chronic wound sites both in the early and late stages [33, 34], suggesting their presence may inhibit wound healing. The absence of NK cells leads to increased local production of monocyte/macrophage and neutrophil chemokines [35]. On the subject of MCs, the number increases in chronic wounds, and their degranulation results in the release of heparin, histamine, and tryptase, further stimulating chronic inflammation [29].

Loots et al. found that chronic diabetic and venous ulcers had more B cells and a reduced CD4/CD8 ratio compared to acute wounds [36]. Chronic wounds are also characterised by functionally impaired T cells [37]. A decrease in the number of B and T cells may be associated with delayed wound healing, but the exact mechanism remains unclear [30]. Similarly, when B cells isolated from wild-type nondiabetic mice were reintroduced into diabetic mice, the wound site was rapidly reduced [38]. Tregs are important for the anti-inflammation transition [39].

Chronic wound fluid contains increased levels of matrix metalloproteinases (MMPs) and proinflammatory cytokines such as TNF*α*, IL-1*β*, and IL-6, as well as decreased growth factor activity [40, 41]. Extended expression of these proinflammatory molecules may lead to a persistent wound environment. Excessive protease activity may potentially destroy growth factors and connective tissue, thereby delaying wound healing [41]. Chronic wounds may be further exacerbated by microbial presence and infection, tissue hypoxia, and other factors at the wound site [14, 32].

There is increasing evidence that acute and chronic musculoskeletal injuries including articular cartilage injury, osteoarthritis, and meniscus injury can be successfully treated with MSCs [42]. We next examine how MSCs accelerate wound closure and whether MSCs influence immune cells to promote the proliferation and tissue remodelling phases in chronic wounds.

### 1.3. The Role of MSCs in Chronic Wounds

Numerous studies have investigated the efficacy of MSC therapy in chronic wounds including chronic ulcers and chronic bacterial infections [54, 55]. MSCs migrate to the site of injury as a result of chemotaxis towards secreted cytokines and growth factors including epidermal growth factor (EGF), platelet-derived growth factor (PDGF), interferon-gamma (IFN-*γ*), interleukin (IL-*β*1), TNF*α*, IL-8, vascular endothelial growth factor-alpha (VEGF-A), insulin-like growth factor (IGF), erythropoietin (EPO), stromal cell-derived factor-1 (SDF-1), and keratinocyte growth factor (KGF) but lower amounts of TGF-*β*1 [56, 57]. MSCs upregulate chemokine receptors such as macrophage-derived chemokine (MDC) receptors CCR2, CCR3, CCR4, IGF-R, and chemokine ligand 5 (CCL5) that enables migration [57]. At the injury site, exposure to proinflammatory cytokines activates the immunosuppressive properties of MSCs [58]. MSCs directly modulate the inflammatory response by decreasing proinflammatory cytokines TNF-*α* and IFN-*γ*, while simultaneously stimulating the production of anti-inflammatory cytokines IL-4 and IL-10, to restart the healing process [14].

Neutrophils in chronic wounds secrete proteases such as elastase and collagenase that destroy growth factors (TGF-*β* and PDGF), break down the extracellular matrix (ECM), and maintain the wound in an inflammatory state [54]. Raffaghello et al. showed that human MSCs inhibit neutrophil apoptosis *in vitro* and *in vivo* via soluble factors such as IL-6 produced by MSCs and not by cell-to-cell contact [59]. Although MSCs appear to promote the accumulation of neutrophils by inhibiting neutrophil apoptosis, they also promote the production of M2 macrophages, thereby promoting wound healing [55].

Mice with chronic tissue injury treated with MSCs express an M2 phenotype, while untreated mice express an M1 phenotype [55]. Zhang et al. [55] demonstrated that MSC soluble factors led to macrophage polarization towards an M2 phenotype *in vitro* and *in vivo*, accelerating wound closure. Mice with an M2 phenotype also secreted more anti-inflammatory Th2-related cytokines such as IL-10 and TGF-*β* and had a lower concentration of Th1-associated cytokines such as IL-6 and TNF-*α* [60]. When mice with chronic wounds were treated with BM-MSCs, they had more macrophages at the wound site [56]. The MSCs in these mice secreted chemoattractants such as macrophage infiltration protein (MIP) and monocyte chemoattractant protein (MCP), thereby promoting the infiltration of macrophages into chronic wounds and support wound healing [56]. MSCs also promote wound healing by modulating the production of effector T cell cytokines. Thus, less of the proinflammatory cytokine IFN-*γ* is produced by Th1 cells and more of the anti-inflammatory cytokine IL-4 from Th2 cells, thereby promoting an anti-inflammatory response [9]. MSCs have TNF receptors that are able to bind TNF-*α* produced by T cells; this then triggers the production of prostaglandin E2 (PGE2). PGE2 acts on T cells and blocks the secretion of TNF-*α* [9]. MSCs promote the arrest of active T cells in the G0/G1 phase of the cell cycle and promote apoptosis, but protect quiescent T cells from apoptosis [9]. High concentrations of MSCs in chronic wounds have also been shown to suppress the proliferation of alloreactive T cells [61]. Similarly, MSCs inhibit the proliferation of B cells by promoting their arrest in the G0/G1 phase of the cell cycle, which further limits the ability of B cells to secrete IgA, IgG, and IgM [61].

MSCs suppress NK cells in various ways including via inhibiting cytokine secretion, cytotoxicity, and proliferation. NK cells secrete IFN-*γ* which induces MSC-derived suppressive factor indoleamine 2,3-dioxygenase (IDO), and in the presence of IL-2, NK proliferation is inhibited [9]. Other MSC soluble factors that suppress the proliferation of NK cells include TGF-*β*, PGE2, and human leukocyte antigen G5 (HLA-G5) [9]. MSCs can inhibit the ability of NK cells to lyse target cells by downregulating surface receptors natural killer group 2 member D (NKG2D), natural killer protein 30 (NKp30), and natural killer protein 44 (NKp44) [9, 62]. Conversely, NK cells can lyse autologous and allogeneic MSCs [63]. Overall, the relative importance of these varied effects on the suppression of NK cells by MSCs culminates in chronic wound healing.

In contrast to chronic wounds that are seemingly stuck in the proinflammatory phase of wound healing and are characterized mostly by an M1 macrophage-associated inflammatory response, the TME is characterised by being in an inflammatory phase of wound healing marked by the presence of an M2 macrophage-associated inflammatory response. In the TME, T cells and NK cells are key players, but MSCs have been shown to inhibit these cells in chronic wounds. To assess the potential role of MSCs in treating cancer, we need to understand how MSCs interact with the immune response in the TME.

### 1.4. Immune Response in the Tumour Microenvironment

The TME is a complex system comprised of numerous cell types in addition to tumour cells, including endothelial cells, pericytes, myofibroblasts, fibroblasts, smooth muscle cells, neutrophils, MCs, T cells, B cells, dendritic cells (DC), macrophages, and NK cells [64]. These cells communicate via autocrine and paracrine signals in the form of cytokines and chemokines. The cross-talk between these cells determines tumour progression or antitumor activity [65]. The immune response to cancer is a dynamic process known as immunosurveillance, which broadly encompasses the concept of immunoediting. Immunoediting is composed of three stages, namely, elimination, equilibrium, and escape [66].

To better understand the effect of MSCs in the TME, this review will use carcinoma as an example to discuss the immune response to cancer. Carcinomas are the most frequent form of malignancy and account for about 90% of all human cancers [64, 67]. Immune cells are directed against cancer in a variety of ways. One of the most important antitumour cell types is cytotoxic CD8+ T cells. Antigen-presenting cells (APCs) such as DCs use their MHC I receptors to present antigens to cytotoxic CD8+ T cells [66, 68, 69]. Upon activation, CD8+ T cells differentiate into cytotoxic T lymphocytes (CTLs). CTLs secrete granzymes and perforins that attack tumour cells resulting in cell lysis [69]. NK cells also recognize antigens of the NKG2D family on tumour cells and release granzymes that kill tumour cells [66]. Antitumour effects are mediated by IFN-*γ* that is secreted by effector cells and that can induce macrophage cytotoxic activity [66, 70].

CD4+ T helper cells can either inhibit or promote tumour cell survival. CD4+ T helper cells are either Th1 or Th2, with each secreting a specific set of cytokines [68, 70]. DCs stimulate Th1 to secrete proinflammatory cytokines including IFN*γ*, TNF*α*, IL-2, and IL-12. These cytokines work together to activate CTLs, M1 macrophages, and NK cells, which increases the presentation of tumour antigens [69–71]. In response, cytokines can induce the upregulation of antigen processing in the proteasome, antigen display cofactors in neoplastic cells, and the expression of MHC I and II [70]. Therefore, Th1 and its secreted cytokines oppose tumorigenesis. On the other hand, Th2 cells secrete progrowth factors IL-4, IL-5, IL-6, IL-10, IL-13, and TGF*β* that inhibit T cell mediated cytotoxicity, cause T cell anergy, and enhance B cell function. Th2 cells further stimulate myeloid-derived suppressor cells (MDSCs) and tumour-associated macrophages (TAMs), which also secrete protumour cytokines (IL-4, IL-5, IL-6, and IL-10) in the TME [71]. Tregs are drawn into the TME through APCs that present self-antigens and inhibit clonal expansion of CTLs [71]. Tregs may also inhibit NK cells, DCs, and B cells through cell-to-cell contact, consequently promoting tumour progression [70, 72]. Elevated numbers of Tregs are associated with a poorer prognosis in many cancers including lung and ovarian cancer, pancreatic ductal adenocarcinoma, and non-Hodgkin's lymphoma [72]. Established tumours contain functionally suppressive Tregs which correlate with advanced tumour stage. Tregs also infiltrate metastatic sites in a manner similar to the TME [72].

Th2 suppress antitumour immunity and improve protumour humoral responses [70, 73]. Part of the humoral response involves a subset of B cells, namely, regulatory B cell (Bregs). Bregs produce IL-10 that suppresses CD4+ T cell cytokine secretion and also suppress CD8+ T cell production of IFN-*γ*. Bregs also encourage conversion of CD4+ into Treg and inhibit NK-mediated immunity, thus promoting tumour progression [73]. The TME facilitates cross-talk between M2, Tregs, and Th2 cells, and this encourages immunosuppression, angiogenesis, and tumour progression [33]. In the TME, neutrophils and MCs exert a proliferative effect [71]. Neutrophils produce high levels of reactive oxygen species (ROS) in the TME, which may cause endothelial damage, DNA mutations that may initiate cancer, cell proliferation, and epithelial-mesenchymal transition (EMT), and favour inflammation [74].

In contrast, although specific lymphocytes are associated with either a pro- or anti-inflammatory response, some anti-inflammatory lymphocytes may inhibit cancer progression. For example, activated antitumour B cells differentiate into clones of plasma cells that produce antibodies specific to the antigens expressed on cancer cells. This leads to antibody-dependent cell-mediated cytotoxicity (ADCC) causing cell lysis via complement activation [68, 70]. In the TME, neutrophils secrete ROS that can eliminate tumour cells and inhibit cancer progression [74]. In summary, neutrophils and B cells may thus either promote or inhibit cancer progression, depending on what they secrete as well as the B cell type involved.

Tumours have other survival strategies including glucose metabolism and tumour acidity. Both pro- and anti-inflammatory cells compete for nutrients in the TME. Tumour cells consume large amounts of glucose which affects the metabolism of other cells in the TME [75, 76]. Lactate production by tumour cells mediated by lactate dehydrogenase A can limit IFN-*γ* production in tumour infiltrating T cells and NK cell activation [75, 76]. Cancer-associated neutrophils use their mitochondrial respiratory capacity to generate ROS, which increases acidity, and in turn, suppresses T cells in the nutrient-depleted TME [75]. Tumour acidity suppresses the activity of DCs, NK, and T cells and supports an M2 phenotype, thus creating a hostile milieu for these cells [75]. Tumour cells in the TME produce stem cell factor (SCF) which can recruit MCs that accumulate near blood vessels. MCs produce proangiogenic factors including VEGF, FGF-2, histamine, heparin, TNF-*α*, and different proteases [77]. These factors are important in initiating the angiogenic switch [77]. MC-mediated ECM remodelling normally seen in wound healing is corrupted in tumour growth, thus promoting tumour migration and metastasis [77].

Apart from proinflammatory leukocytes that support tumour growth, elements of the coagulation pathway further exacerbate the already compromised TME. Tissue factor (TF), which initiates coagulation, is expressed by tumour cells and is increased in breast, glial, colon, and lung cancers. TF expression has been associated with increased vascular density, tumour grade, and a worse prognosis [78, 79]. Platelets and thrombin within the TME are associated with tumour growth, angiogenesis, and metastasis [79]. This is because platelets release growth factors such as TGF-*β* which is a key mediator of angiogenesis in the TME [25, 80]. It is noteworthy that thrombosis is the second leading cause of death in cancer [78], and understanding its role is therefore crucial. Another factor that contributes to tumorigenesis is whether the microenvironment is acidic or hypoxic, as this compromises the antitumour activity of immune cells [75]. It is therefore important to examine other factors that contribute to tumorigenesis and metastasis to obtain a better understanding of the complex nature of the TME.

### 1.5. The Role of MSCs in the TME

MSCs migrate to tumour sites in breast, colon, ovarian, brain, hepatocellular, and lung carcinomas as well as melanoma [17, 81, 82]. This has raised the possibility that MSCs might serve as a potential delivery vehicle for anticancer drugs [83–85]. The ability of MSCs to influence the immune response in chronic wounds, thereby promoting healing, has exciting prospects. It is important therefore to understand how MSCs may affect various cell types including leukocytes in the TME.

MSCs affect cytokine secretion, differentiation, the phenotype of mature and immature monocyte-derived DCs, as well as immunostimulatory activity such as the induction of T cell activation [9, 86]. Additionally, MSCs reduce the expression of costimulatory molecules such as CD80 and CD86 [9]. Hence, MSCs inhibit the function of DCs leading to immunological tolerance [62]. MSCs also induce regulatory APCs that generate elevated levels of IL-10 and suppress T cells [9]. Since different cancers behave differently, it is evident that the influence of MSCs on various cancers will differ. For this reason, this section will focus on studies that have looked at the influence of MSCs on breast cancer.

Patel et al. [62] demonstrated that MSCs interact with lymphocytes that inhibit breast cancer cells in a manner similar to chronic wounds. The presence of MSCs suppresses NK cells and diminishes CTL responses. Consequently, less granzyme is produced for tumour cell killing. Additionally, MSCs produce IL-10 which inhibit T cell proliferation [62]. When MSCs were added to breast cancer cells, there was less production of Th1 type cytokines IFN-*γ* and TNF-*α*, while Th2 type cytokines were significantly increased [62]. MSCs also secrete TGF-*β*1 which increases Tregs production [62]. An *in vivo* breast cancer study demonstrated that tumour-derived MSCs (T-MSCs) and TNF-*α*-activated MSCs increased neutrophil tumour infiltration. More neutrophils were also observed in the blood and primary tumour [87]. Additionally, TNF-*α*-activated MSCs mimicked T-MSCs by promoting tumour growth and breast cancer metastasis to the lung [87].

There are other ways in which MSCs promote cancer progression. In a breast cancer study, the presence of MSCs in the TME acted as a stimulus to induce EMT [18]. EMT promoted cancer progression including metastasis to other tissues [18]. Cancer cell survival is also promoted by the ability of MSCs to differentiate into cancer-associated fibroblasts (CAFs) [88]. CAFs promote Treg capacity to inhibit effector T cell proliferation [89]. MSCs secrete exosomes that enter dormant breast cancer cells (BCCs) and initiate cycling thus increasing BCCs [90].

An *in vitro* study showed that MSCs inhibit cell cycle progression in breast cancer cells and promote apoptosis by downregulating the *Stat3* signalling pathway [23]. This study did not describe how MSCs affect lymphocyte function. Leng et al. [22] demonstrated that UC-MSCs loaded with a suicide gene (herpes simplex virus truncated thymidine kinase) inhibited tumour growth and increased apoptosis, but complete regression was not achieved [22]. An *in vitro* study using the immortalized MSC line RCB2157 cocultured with MDA-MB-231 and T47D cells further demonstrated that MSCs are antimetastatic and antitumorigenic. In the first 24 hours, MSCs secreted MMPs that have a prometastatic effect. After 5 days in culture, MSCs secreted tissue inhibitors of metalloproteinase-1 and -2 (TIMP-1 and TIMP-2) which have an antimetastatic effect [24]. Another study demonstrated that even in the presence of MSCs, CD8+ T cells, DCs, and CD4+/Foxp3 Treg remained normal [91]. Similar studies have observed that MSCs inhibit cancer progression both *in vivo* and *in vitro* [91–93]. Several factors including specific pathways such as stat3 signalling, the source of MSC, nature of MSCs (modified or naïve), choice of mouse models (syngeneic, allogeneic, and xenogeneic), types of breast cancer cell lines, and MSC secreted molecules such as exosomes appear to be responsible for the inhibitory effects on cancer [7, 91–94].

MSCs lack costimulatory molecules (CD40, CD80, and CD86) and MHC II; however, these molecules can be induced when MSCs are exposed to IFN-*γ*. When IFN-*γ* is upregulated, MHC II expression is increased and accessory adhesion molecules such as intracellular adhesion molecule 2 (ICAM-2) and vascular cell adhesion molecule 1 (VCAM-1) are expressed [9, 63]. This is important because their expression allows MSCs to act as APCs to memory T cells [9]. This may also be the intercellular contact needed for MSCs to exert a cytotoxic effect on NK cells [9], thereby decreasing NK cells with antitumour activity.

MSCs thrive in hypoxic (2-9% O2) conditions such as those found in the TME, and this environment therefore resembles their natural niche (1-5% O2). When expanded *in vitro*, MSCs are grown in 20% oxygen, and this may cause DNA damage and early senescence [95, 96]. Consequently, *in vivo* administration of possibly compromised MSCs may not portray a true reflection of their influence on tumours under natural conditions *in vivo*. Similarly, in *in vitro* studies when MSCs were cocultured with a head and neck squamous cell carcinoma cell line (FaDu), cancer cell viability and proliferation decreased when incubated in conditioned media that was derived from MSCs grown under hypoxic conditions. Levels of anti-inflammatory cytokines such as IL-4, IL-5, and IL-10 are reduced, and IL-6 is increased in this conditioned medium [97]. This data suggests that despite the fact that MSCs are expanded under normoxic conditions, they may reestablish homeostasis in the hypoxic TME and still have an effect on cancer cells. However, further investigation of the ability of MSCs to reestablishing homeostasis is needed.

The effect of MSCs on the immune response in chronic wounds is similar to that observed in the TME in that they exhibit immunosuppressive characteristics [62, 87]. MSCs do this by secreting immunosuppressive factors including IDO and NO that suppress lymphocyte function, thereby promoting tumour progression and metastasis [87]. The effect of MSCs on different cancer types varies since different cancers behave differently. MSCs inhibit the growth of human glioma cells, while in human colorectal cancer cells, tumour growth is initiated and promoted [98]. The TME has more lymphocytes and cytokines that promote cancer growth than inhibitory factors. However, there is also evidence that MSCs inhibit tumour progression and metastasis. Therefore, the presence of MSCs in the TME could either be beneficial or harmful to the host as there is no clear evidence in support of one effect over the other.

### 1.6. Summary and Key Findings

This review has discussed the role of MSCs and their influence on the immune response in chronic wounds and the TME. Chronic wounds are in a continuous proinflammatory state due to the constant presence of immune cells whose function is impaired in the wound environment. These cells include neutrophils, macrophages, B cells, and T cells. There is a decrease in the number of lymphocytes while Treg increase and the transition from M1 to M2 is delayed. MSCs induce a transition to an M2 type inflammatory response and modulate effector T cell cytokines which leads to reduced proinflammatory cytokine production and an increase in anti-inflammatory cytokines. MSCs promote the arrest of T and B cells at G0/G1 in the cell cycle, thereby promoting T cell apoptosis and limiting antibody secretion by B cells. MSCs also suppress NK cell proliferation and cytokine secretion, which under normal circumstances would enable chronic wounds to heal.

Th1 via proinflammatory cytokines activate CTLs, DCs, and NK cells in the TME to attack tumour cells. In contrast, Th2 produce anti-inflammatory cytokines that inhibit T cell cytotoxicity and increase B cell function. Tregs inhibit CTLs, DCs, NK, and B cells while Bregs suppress CD8+ T cells and encourage conversion of CD4+ T cells to Tregs. All of the above support tumour progression and metastasis. The presence of MSCs in the TME encourages tumour progression via several mechanisms: (a) monocytes fail to differentiate into DCs, delaying the activation of T cells; (b) MSCs inhibit T cell proliferation and suppress NK and CTL responses; (c) MSCs induce Th2 cytokine production and decrease Th1 cytokine production; (d) MSCs induce EMT and differentiate into CAFs that secrete angiogenic growth factors. Together, these observations suggest that MSCs support cancer growth and metastasis, and this is based primarily on the immunosuppressive properties of MSCs. Conversely, other studies have demonstrated that MSCs can inhibit tumour growth and metastasis by (a) downregulating the *Stat3* pathway and (b) MSCs producing TIMP-1 and TIMP-2.

Despite MSCs exhibiting similar immunosuppressive characteristics in both chronic wounds and the TME, several studies have observed that MSCs inhibit cancer progression. In chronic wounds, the application of MSCs is beneficial while in the TME, their application could be either beneficial or detrimental. Various factors such as immunosuppression, glucose metabolism, and Th2 associated leukocytes and cytokines may result in a net effect in which tumour progression outweighs inhibition. The use of MSCs in the clinical setting for therapeutic purposes unrelated to cancer should be approached with this in mind and requires long-term follow-up/monitoring as MSCs may promote cancer cell survival and stimulate the progression of a latent tumour.

## 2. Conclusion

The TME is similar to chronic wounds in that the phases of healing, inflammation, and proliferation are either hijacked or stalled. In chronic wounds, the constant inflammatory state can be corrected by applying MSCs to downregulate T cells, NK cells, and B cells, resulting in enhanced wound healing. In the TME, different lymphocyte subtypes promote rather than inhibit cancer progression. Tumour cells also secrete immunosuppressive molecules and compete for nutrients. All these factors contribute to the complex nature of the TME. While a few studies have suggested that MSCs inhibit cancer progression, a greater number suggest that MSCs increase tumour progression and metastasis. Hence, based on the available literature on the role of MSCs in the TME, the use of MSCs for the treatment of cancer is not supported, as MSCs in the TME suppress inflammation and the immune response that is required for wound healing.

## Data Availability

The data supporting the results cited in the text can be found in the relevant articles cited in the References.

## Abbreviations

ADCC: Antibody-dependent cell-mediated cytotoxicity
APC: Antigen-presenting cell
AT-MSCs: Adipose tissue mesenchymal stem cells
BCCs: Breast cancer cells
bFGF: Basic fibroblast growth factor
BM-MSCs: Bone marrow mesenchymal stem cells
Bregs: Regulatory B cells
CAFs: Cancer-associated fibroblasts
CCL 5: Chemokine ligand 5
CCR: C-C chemokine receptor
CD: Cluster of differentiation
CTLs: Cytotoxic T cells
DCs: Dendritic cells
DNA: Deoxyribonucleic acid
ECM: Extracellular matrix
EGF: Epidermal growth factor
EMT: Epithelial-mesenchymal transition
EPO: Erythropoietin
FGF: Fibroblast growth factor
GM-CSF: Granulocyte-macrophage colony-stimulating factor
HLA-DR: Human leukocyte antigen-DR isotype
ICAM-2: Intracellular adhesion molecule 2
IFN-*γ*: Interferon-gamma
IDO: Indoleamine 2,3-dioxygenase
Ig: Immunoglobulin
IGF: Insulin-like growth factor
IGF-R: Insulin-like growth factor 1
IL: Interleukin
KGF: Keratinocyte growth factors
M1: Proinflammatory macrophages
M2: Antimacrophages
MCs: Mast cells
MCP-1: Monocyte chemoattractant protein*-*1
MDC: Macrophage-derived chemokine
MDSCs: Myeloid-derived suppressor cells
MHC: Major histocompatibility complex
MIP: Macrophage infiltration protein
MMPs: Matrix metalloproteinases
MSCs: Mesenchymal stem/stromal cells
NK: Natural killer
NKG2D: Natural killer group 2 member D
NKp: Natural killer protein
NO: Nitric oxide
O2: Oxygen
Oct4: Oct-4 (octamer-binding transcription factor 4)
p53: Tumor protein P53
PDGF: Platelet-derived growth factor
PGE2: Prostaglandin E2
ROS: Reactive oxygen species
rTEM: Transendothelial migration
SCF: Stem cell factor
SDF-1: Stromal cell-derived factor-1
Sox2: SRY (sex determining region Y)-box 2S
Stat3: Signal transducer and activator of transcription *3*
TAMs: Tumour-associated macrophages
TGF-*β*: Transforming growth factor-beta
TF: Tissue factor
Th: T helper
TIMP: Tissue inhibitors of metalloproteinases
T-MSCs: Tumour-derived mesenchymal stem cells
TME: Tumour microenvironment
TNF*α*: Tumour necrosis factor alpha
Treg: Regulatory T cells
VCAM-1: Vascular cell adhesion molecule 1
VEGF: Vascular endothelial growth factor
UC: Umbilical cord
UCB: Umbilical cord blood.
